# Adverse Childhood Experiences and Risk of Subsequently Engaging in Self-Harm and Violence towards Other People—“Dual Harm”

**DOI:** 10.3390/ijerph17249409

**Published:** 2020-12-15

**Authors:** Matthew J. Carr, Sarah Steeg, Pearl L. H. Mok, Carsten Bøcker Pedersen, Sussie Antonsen, Nav Kapur, Roger T. Webb

**Affiliations:** 1 NIHR Greater Manchester Patient Safety Translational Research Centre, University of Manchester, Manchester M13 9PL, UK; matthew.carr@manchester.ac.uk (M.J.C.); nav.kapur@manchester.ac.uk (N.K.); roger.webb@manchester.ac.uk (R.T.W.); 2 Centre for Pharmacoepidemiology and Drug Safety, Division of Pharmacy & Optometry, University of Manchester, Manchester M13 9PT, UK; pearl.mok@manchester.ac.uk; 3 Centre for Mental Health and Safety, Manchester Academic Health Science Centre, University of Manchester, Manchester M13 9PL, UK; 4 Centre for Integrated Register-based Research, National Centre for Register-based Research, Business and Social Sciences, Aarhus University, Fuglesangs Allé 26, Building 2640, 8210 Aarhus V, Denmark; cbp@econ.au.dk (C.B.P.); sa@econ.au.dk (S.A.); 5 Greater Manchester Mental Health NHS Foundation Trust, Manchester M25 3BL, UK

**Keywords:** self-harm, dual harm, violence, childhood adversity, substance misuse

## Abstract

The etiology of “dual harm” (the co-occurrence of self-harm and externalized violence in the same individual) is under-researched. Risk factors have mostly been investigated for each behavior separately. We aimed to examine adversities experienced between birth and age 15 years among adolescents and young adults with histories of self-harm and violent criminality, with a specific focus on dual harm. Three nested case-control studies were delineated using national interlinked Danish registers; 58,409 cases in total aged 15–35 were identified: 28,956 with a history of violent criminality (but not self-harm), 25,826 with a history of self-harm (but not violent criminality), and 3987 with dual-harm history. Each case was matched by date of birth and gender to 20 controls who had not engaged in either behavior. We estimated exposure prevalence for cases versus controls for each of the three behavior groups, and incidence rate ratios (IRRs). Experiencing five or more childhood adversities was more prevalent among individuals with dual-harm history (19.3%; 95% CI 18.0, 20.8%) versus self-harm (10.9%; 10.5, 11.3%) and violence (11.4%; 11.0%, 11.8%) histories. The highest IRRs for dual harm were linked with parental unemployment (5.15; 95% CI 4.71, 5.64), parental hospitalization following self-harm (4.91; 4.40, 5.48) or assault (5.90; 5.07, 6.86), and parental violent criminality (6.11; 5.57, 6.70). Growing up in environments that are characterized by poverty, violence, and substance misuse, and experiencing multiple adversities in childhood, appear to be especially strongly linked with elevated dual-harm risk. These novel findings indicate potential etiologic pathways to dual harm.

## 1. Introduction

There is a considerable body of published research concerning the characteristics of young people who have harmed themselves, and the determinants of this behavior are well known [1,2,3,4,5,6]. This extensive evidence-base enables services to be designed to meet the needs of individuals who present to services following self-harm. Similarly, risk factors for violent behavior in the general population [7,8,9] and in people with a history of mental illness [10] are well established. However, evidence concerning the etiology of both behaviors co-occurring in the same individuals (which from now on we refer to as “dual harm”) is sparse [11]. Estimates of the prevalence of dual harm in the general population are lacking, though one study of adolescents suggested that 2.9% had engaged in both self-harm and violent crime. [12] However, reported dual-harm prevalence values will vary considerably according to the definitions used for the two overlapping behaviors. Furthermore, prevalence is notably higher in prison settings, with one study finding that 11% of male prisoners had a history of dual harm [13]. Young people who harm themselves and also subject other people to violence have recently been found to be far more likely to die from external causes, especially by accidental poisoning, before the age of 35, and to have more severe psychopathology than those with a history of only one of these two damaging behaviors [14,15].

There is increasing understanding of risk factors that are common to people who self-harm and those who engage in interpersonal violence. For example, almost all types of parental mental illness were found to be risk factors for self-harm and violent criminality among cohort members in a Danish register-based study that examined both behavioral outcomes in the same national birth cohort [8]. Low parental income and parental death during childhood have also been linked to both self-harm and violent criminality in other national register-based studies conducted in Denmark [9,16]. In a US survey of the adult general population [17], impulsivity and childhood maltreatment independently predicted self-inflicted harm and violence inflicted upon others. However, this study and others too have also found some differences in the strengths of association for self-harm versus externalized violence. For example, Harford et al. [15] reported significant differences in the types of mental disorders associated with violence risk versus self-harm risk. In that study, risk of externalized violence was more strongly associated with substance use disorders and personality disorders, whilst self-harm was more strongly associated with mood and anxiety disorders. Harford et al. [18] found that alcohol and drug use disorders, mood disorders, and posttraumatic stress disorder were significantly associated with dual harm. However, few investigators have previously reported characteristics of people engaging in dual harm, with most studies to date examining the two behaviors separately.

There is very little understanding of early life risk factors for the co-occurrence of dual harm. Recognition and treatment of early trauma is necessary to address the causes of externalized violence [19]. Unresolved traumatic experiences in childhood have also been identified in the trajectory to self-harm [20]. Adolescence is a key phase for the emergence of self-harming and violent behaviors [21]. A recent study examined risk factors for self-harm and violence in adolescence [12]. Poor self-control was reported to distinguish adolescents in the dual-harm group from those who had self-harmed but had not inflicted violence on other people. Furthermore, adolescents engaging in dual harm were more likely to have experienced childhood maltreatment and victimization and had higher rates of alcohol and substance misuse. Relationships across a broader array of adverse childhood experiences in the general population and dual-harm risk in young adulthood have not yet been examined. Furthermore, much of the previous research on risk factors has been conducted in clinical samples or forensic settings rather than in the general population [22,23]. 

Understanding the characteristics of people in the general population who engage in dual harm is vital for addressing the specific needs of this risky and vulnerable group. A systematic review of studies that have examined the relationship between violence and self-harm found that the occurrence of either of these harmful behaviors resulted in increased risk of the other one also occurring [11]. That study’s authors concluded that further research was needed to understand more about individuals who engage in both damaging behaviors, including a greater understanding of risk factors for dual harm. Identifying the specific determinants of dual harm is key to developing strategies for reducing its prevalence and providing effective interventions to prevent dual harm or ameliorate its impact. Given the distinct risk profiles of people who engage in dual harm as regards a markedly elevated risk of dying by external causes [14], we hypothesized that the etiologic profile of this group would also be distinct from those of the two “single harm” groups. The first aim of our study was to utilize national interlinked registry data from Denmark to examine the prevalence of a range of personal and family adversities experienced in childhood among adults aged 15–35 years who had engaged in: (1) self-harm but not violent criminality; (2) violent criminality but not self-harm; (3) dual harm—i.e., both harmful behaviors. Our second aim was to examine the relative strength of the associations between these adverse childhood experiences and self-harm, violent criminality and dual harm. 

## 2. Materials and Methods 

From a national birth cohort we delineated three nested case-control studies, with cases defined as (i) persons with an episode of hospital-treated self-harm but no record of violent criminality, (ii) persons with a record of committing violent crime but not engaging in self-harm, and (iii) those with a history of both harmful behaviors—dual harm. Self-harm episodes and violent criminal offending were identified if they occurred after cohort members’ 15th birthdays. Each case was matched to twenty controls who had no history of either self-harm or violent criminality, selected randomly from all eligible individuals in each risk set. Cases and controls were matched by gender and by date of birth using incidence density sampling procedures [24,25]. Matching on the date of birth ensured that each case and their matched controls were exactly the same age, which controlled for potential confounding age and cohort influences. Individuals could be selected as controls for more than one case and could also be sampled for more than one of the three case-control studies. We followed STROBE (Strengthening the Reporting of Observational Studies in Epidemiology) guidelines [26].

Each Danish resident is assigned a unique personal identification number, a comprehensive system that enables accurate linkage between multiple administrative registers with complete national population coverage. Each of the three case-control datasets was nested in a cohort of persons born in Denmark to native Danish parents during 1980–2000 and who were alive and residing in the country on their 15th birthdays (N = 1.08 million). Measurement of exposure status began at birth and ended on cohort members’ 15th birthdays. Self-harm episodes and violent crimes were measured from age 15 onwards. Therefore, all cohort members were aged between 15 and 35 years during the study’s observation period. 

We were primarily interested in dual harm, which we defined as having a history of both self-harm and violent criminality after reaching age 15. We also examined self-harm (without violent criminality) and violent criminality (without self-harm) as “single harm” comparator outcomes. From 1990, hospital-treated self-harm episodes included those resulting in admissions to general hospitals and psychiatric units, and from 1994 onwards it also included general hospital emergency department presentations and episodes treated in psychiatric unit outpatient clinics. These episodes were identified from the National Patient Register [27] and from the Psychiatric Central Research Register [28] by applying a commonly used coding algorithm derived in a previous study (Box S1, Supplementary Materials) [29]. This definition of “self-harm” includes intentional nonfatal acts, with or without suicidal intent and including self-poisoning and self-injury [21,30,31]. Very low self-harm incidence prior to the 1990s indicated that self-harm may not have been fully recorded in the registers in earlier years, so we restricted study cohort to individuals born from 1980 and onwards. Information regarding violent crimes was extracted from the National Crime Register [32]. In addition to physical and sexual assault, we included threats of violence, organised crime such as human trafficking, and public order offenses such as rioting [14]. We applied the date when the criminal act was recorded as occurring. For 0.3% of all violent crimes, the date when the offense was committed was not registered, so we applied the conviction date instead. We were interested in self-harm, violent criminality and dual-harm outcomes following exposures between birth and 15^th^ birthday, on the basis that the minimum age for criminal responsibility in Denmark is 15 years. Therefore, we included self-harm episodes and violent offenses from individuals’ 15th birthday and onwards.

Adverse experiences occurring before a cohort members’ 15th birthday were examined, including factors pertaining to the individual (personal risk factors) and those relating to their parents and family environment. Personal exposures were hospitalization following self-harm, assault, or serious accident. Family environment factors included: parental unemployment, low parental income and low parental educational attainment, younger maternal age, older paternal age, residential transience, sibling death, parental death, parental hospitalization due to self-harm, assault and serious accident, parental mental illness, parental violent criminality, and child–parent separation. The exposures were selected based on data availability within the national registers for factors related to childhood and family experiences.

We fitted conditional logistic regression models to estimate relative risks for the three outcome categories of interest: (1) self-harm (but not violent criminality); (2) violent criminality (but not self-harm); and 3) dual harm (self-harm + violent criminality)—separately within each respective nested case-control study. Cases in each outcome category were compared to controls with no record of self-harm or violent criminality as regards exposure prevalence for each childhood adversity examined. In the nested case-control study design, the exposure odds ratio estimate is equivalent to the incidence rate ratio, IRR (i.e., relative risk). All analyses were performed using Stata Release 15 [33]. 

This study did not require approval from the Danish National Committee on Health Research Ethics because it was conducted solely using registry data. Approval to conduct this study was granted by the Danish Data Protection Agency, the Danish Health Data Authority, and Statistics Denmark.

## 3. Results

### 3.1. Description of the Study Cohort

The national birth cohort from which the three nested case-control studies were delineated included 1,226,589 Danish people. A total of 58,409 cases were identified, including 28,596 people with a history of violent criminality (but not of self-harm), 25,826 who had previously self-harmed (but had no history of violent criminality), and 3987 with dual harm; i.e., a record of both harmful behaviors. 

### 3.2. Prevalence of Personal and Family Risk Factors among Cases and Controls with Histories of Self-Harm, Violent Criminality and Dual Harm

The prevalence of most childhood risk factors was not significantly higher in the dual-harm group compared to the single harm groups (Table 1). Two exceptions were hospitalization due to assault (1.2%, 95% CI 0.9, 1.6%), which was at least twice as prevalent than among those with a history of violent criminality (0.6%, 0.5, 0.7%) or self-harm (0.4%, 0.3, 0.5%), and hospitalization following a serious accident, experienced by 17.9% (16.6, 19.3%) of those with dual-harm history compared to 15.8% (15.4, 16.3%) among those with a history of violence and 13.7% (13.2, 14.1%) of those with a history of self-harm. 

Most of the family risk factors that we measured were more prevalent in the dual-harm group than in either of the single-harm groups, including parental unemployment, low parental income, and low parental educational attainment, younger maternal age, residential transience, parental death from external causes, hospitalization due to self-harm, assault or serious accident, parental substance misuse disorder and other mental disorder, parental violent criminality and child–parent separation (Table 1). The prevalence of experiencing a greater number of risk factors was also markedly higher in the dual-harm group compared to either of the two single-harm groups (Table 2); 19.3% (18.0, 20.8%) in the dual-harm group experienced five or more childhood adversities compared to 10.9% (10.5, 11.3%) in the self-harm group and 11.4% (11.0, 11.8%) who had subjected other people to violence.

### 3.3. Relative Risks for Violent Criminality, Self-Harm and Dual Harm by Exposure Group

The incidence rate ratios (IRRs) for self-harm and violent criminality were markedly raised for young people experiencing hospitalization for assault and serious accident (Table 3). The risks of dual harm were additionally raised for those experiencing hospitalization for self-harm and were highest following assault. Similarly, raised IRRs for self-harm and violence were observed for those experiencing most of the parental and family risk factors examined, with excess risks found for dual harm. The highest IRR values for dual harm were with the following parental risk factors: unemployment (5.15, 95% CI 4.71, 5.64), substance misuse disorder (5.09, 95% CI 4.61, 5.63), violent criminality (6.11, 95% CI 5.57, 6.70), and hospitalization following self-harm (4.91, 95% CI 4.40, 5.48) or assault (5.90, 95% CI 5.07, 6.86). These IRRs were all higher for dual harm than for either of the two single harm groups. 

### 3.4. Relative Risks of Violent Criminality, Self-Harm and Dual Harm by Number of Adverse Childhood Experiences

**Figure 1:**
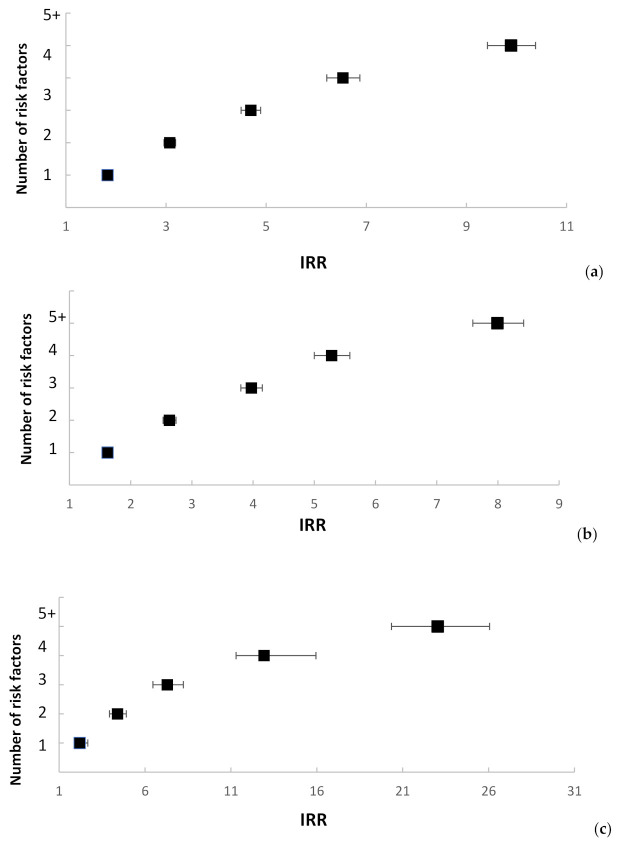
Incidence Rate Ratios (IRRs) ^1^ by number of adverse childhood experiences. (**a**) Incidence Rate Ratios (IRRs) ^1^ for violent criminality by number of adverse childhood experiences ^2^; (**b**) Incidence Rate Ratios (IRRs) ^1^ for self-harm by number of adverse childhood experiences ^2^; (**c**) Incidence Rate Ratios (IRRs) ^1^ for dual harm by number of adverse childhood experiences ^2^; ^1^ Reference categories for IRRs: cohort members without history of any of the measured risk factors; ^2^ Number of adverse childhood experiences does not correspond exactly to the sum of the individual exposure variables. For instance, the parental separation exposure variable can take values 0 (no separation = reference category), 1 (separation = exposed), or a missing category taking the value 2 (one or more parents died before a separation could occur). In Table 2, comparison between 1 and 0 (separation vs. no separation) is compared. However, for the “Number of adverse childhood experiences” variable, it was necessary to combine categories 0 and 2 and reduce the individual exposures to binary variables (i.e., experienced the adversity vs. did not experience the adversity).

Incremental increases in the IRRs for violent criminality, self-harm, and dual harm were observed as the number of adversities that individuals were exposed to during their childhood increased (Figure 1). Whilst this pattern was seen for all three harmful behavior groups, the risk was more than doubled for the dual-harm group. For example, risks of violent criminality and self-harm were between 8 and 10 times higher for those individuals who had experienced 5 or more types of adverse childhood adversity, whereas dual-harm risk was 23 times higher in the presence of this greater number of adversities during their upbringing (Figure 1). 

## 4. Discussion

In this study, we found that young people with histories of violent criminality, self-harm and dual harm had raised prevalence of the adverse childhood experiences investigated. However, those who had engaged in dual harm had considerably higher prevalence for some of the examined risk factors, and they were also much more likely to have experienced multiple adversities whilst they were growing up compared to one or two such risk factors, versus their peers with single-harm histories. In terms of personal adverse experiences, prior hospitalization for assault predicted a particularly large risk elevation for subsequent dual harm. With respect to family adversity, we observed a substantial excess risk of dual harm among children whose parents were unemployed or had been diagnosed with a substance misuse disorder, who had engaged in violent criminality, or who had been hospitalized following self-harm or assault.

We observed the highest risks of dual harm among children who had been hospitalized for assault during childhood or had grown up with parents who had subjected other people to violence, who had been the victim of assault or who had engaged in self-harm requiring hospital treatment. These experiences of violence victimization and exposure to parental violence were stronger predictors than other adverse experiences such as suffering a serious accident or the death of a parent from natural causes. Whilst we cannot infer direct causality between childhood exposure to interpersonal violence and subsequent elevated dual-harm risk, it is a marker that could potentially help explain the pathways to dual harm, which is currently a poorly understood phenomenon. Parental and childhood adversity, and especially the accumulation of multiple adverse experiences, increase self-harm risk in young people [2,34]. Similarly, a large international study found that a range of childhood adverse experiences were strongly associated with all classes of mental disorders in adulthood [35]. A number of mechanisms are likely to be driving the higher likelihood of poor outcome. Parental trauma or adversity may contribute to the development of maladaptive psychological and behavioral processes, as well as biological changes induced by childhood trauma, which persist into adulthood. Parental modelling, whereby social norms and behaviors are transmitted to offspring during childhood, may also influence responses to stress in adulthood [36]. Finally, effects of childhood adversity are exacerbated by low socioeconomic position [9,34].

Our findings build upon a number of studies. Investigators in the USA found that experiencing multiple and persistent adversities between ages 4 and 16 was associated with greater use of healthcare services and poorer self-reported health at age 18 [37]. Parental death inferred increased risk of both self-harm and violent criminality [16], but we have shown that the risk of dual harm among children who lost a parent is even greater—especially death from external causes. Webb et al. [38] found that rates of self-harm and violent criminality were considerably raised among adults aged 35 years and under who had been hospitalized for self-harm or assault before the age of 15. In the present study, hospitalization following assault was the only personal adverse experience to infer an excess risk of dual harm over and above the risks of self-harm and violence examined as discrete “single harm” outcomes. 

In a recently published study examining early life predictors of dual harm in adolescents [12], those who engaged in dual harm were more likely to self-harm with higher lethality and more aggressive methods, and had higher rates of psychiatric comorbidity that those who self-harmed and did not act violently toward other people. This is concerning, because individuals engaging in dual harm may not receive any additional mental health treatment compared to those who self-harm, despite the additional difficulties that they face [12]. However, unlike the present study, in which we only included self-harm episodes resulting in hospital attendance, Richmond-Rakerd et al. also included self-harm episodes for which no treatment was received, which is less likely to involve self-poisoning [39]. They also studied a younger cohort; self-harm in older adolescents and young adults tends to be more severe and associated with mental health problems than in younger adolescents [40,41]. Potentially, the needs of individuals engaging in dual harm within an older cohort aged between 15 and 35 are even greater. 

Attempted suicide in under-24-year-olds was found to be associated with increased risks of violent crime and intimate partner abuse perpetration into mid-life, as well as a range of other damaging social and health outcomes [42]. This highlights the long-term consequences for people who have self-harmed; while most adolescents who have self-harmed experience resolution of their distress [43], a minority will go on to experience considerable social adversity and enduring mental health problems. Because of the elevated risk of suicide among adolescents with a history of childhood adversity and externalized violence [44], future research should consider risk factors associated with suicide in this vulnerable group. Decker et al. [45] have argued that an integrated approach to preventing suicide and externalized violence is needed. 

This is the first study to examine dual harm in the context of multiple psychosocial adverse events spanning birth to 15 years of age and follow-up into mid-adulthood, highlighting the enduring consequences of adverse childhood experiences. We utilized national interlinked Danish registry data, enabling the examination of multiple adverse childhood experiences with minimal risk of recall and reporting bias. A limitation of our study was that we could only identify persons who presented to hospital after harming themselves, ascertaining a relatively small proportion of all self-harm episodes that occur in the community [39]. Individuals not seeking medical treatment following a self-harm episode, or those treated in primary care without hospital attendance, or treated before their 15th birthday, were not included. Likewise, we included violent criminality only without capturing any information on episodes of interpersonal aggression and violence that do not result in criminal conviction. Therefore, our findings may not be generalizable to self-harm and violence of lower severity, and consequently, the dual-harm group may be considerably larger in size than what we have reported. Furthermore, certain violent crimes, such as intimate partner violence and sexual violence, are less likely to be known to the authorities and result in conviction. We were unable to study people born before 1980. Finally, our study cohort did not include information for people without at least one native-born Danish parent, due to incomplete information on parental risk factors for these individuals. 

Our findings have a number of implications. People engaging in dual harm should be identified as early as possible in educational, healthcare, social services, and criminal justice settings. Our findings highlight and further emphasize the need for thorough assessment and early intervention for people presenting to hospital with self-harm [46], including consideration of people’s family environment and their psychosocial needs. Identifying co-occurring violence among this group should be routinely included in assessments. Furthermore, young people engaging in interpersonal violence who have also self-harmed require interventions that recognize their especially high prevalence of multiple family adversities as well as their distinct elevated mortality risk profile [14].

## 5. Conclusions

Children growing up alongside violence, poverty, and substance misuse are considerably more likely to engage in dual harm during late adolescence and early adulthood. Experiencing multiple adversities during childhood was associated with elevated risks of violent criminality and self-harm and an even higher dual-harm risk. Further research should examine the relationship between early life predictors of dual harm as certain combinations of experiences may be particularly harmful. Potentially protective factors should also be examined.

## Figures and Tables

**Table 1:** Exposure prevalence of adverse childhood experiences among cases vs. controls for the 3 behavioral outcome categories.

Childhood Adversities	1. Violent Crime (but Not Self-Harm)				2. Self-Harm (but Not Violent Crime)				3. Dual Harm			
	Cases		Controls		Cases		Controls		Cases		Controls	
	(*N* = 28,596)		(*N* = 571,920)		(*N* = 25,826)		(*N* = 516,520)		(*N* = 3987)		(*N* = 79,740)	
	*n*	%	*n*	%	*n*	%	*n*	%	*n*	%	*n*	%
Personal risk factors												
Hospitalization due to												
Self-harm ^‡^	432	1.5	2997	0.5	925	3.6	3299	0.6	132	3.3	446	0.6
Assault ^‡^	173	0.6	712	0.1	109	0.4	542	0.1	47	1.2	127	0.2
Serious accident ^‡^	4522	15.8	62,868	11.0	3533	13.7	49,056	9.5	715	17.9	8498	10.7
Parental/family risk factors												
Sociodemographic factors												
Unemployment ^ƚ^	3545	12.4	20,776	3.6	2847	11.0	19,361	3.7	697	17.5	3151	4.0
Low educational attainment—both parents ^ƚ^	6535	22.9	61,360	10.7	5222	20.2	55,101	10.7	1116	28.0	9053	11.4
Younger maternal age	2268	7.9	15,336	2.7	1770	6.9	14,061	2.7	438	11.0	2449	3.1
Older paternal age	1529	5.3	35,531	6.2	1552	6.0	32,098	6.2	205	5.1	4826	6.1
Residential transience ^‡^	4004	14.0	29,215	5.1	3341	12.9	27,139	5.3	791	19.8	4269	5.4
Adverse events												
Sibling death ^‡^	905	3.2	13,496	2.4	835	3.2	12,094	2.3	159	4.0	1901	2.4
Parental death												
External causes ^‡^	701	2.5	4986	0.9	602	2.3	4710	0.9	159	4.0	749	0.9
Natural causes ^‡^	911	3.2	11,758	2.1	780	3.0	10,888	2.1	147	3.7	1717	2.2
All causes	1601	5.6	16,660	2.9	1367	5.3	15,538	3.0	302	7.6	2453	3.1
Hospitalization due to												
Self-harm ^‡^	1995	7.0	13,252	2.3	1966	7.6	12,171	2.4	437	11.0	1946	2.4
Assault ^‡^	1085	3.8	5119	0.9	798	3.1	4935	1.0	227	5.7	808	1.0
Serious accident ^‡^	5661	19.8	75,776	13.2	4826	18.7	68,567	13.3	924	23.2	10,655	13.4
Mental illness diagnosis												
Substance misuse disorder ^‡^	2422	8.5	16,268	2.8	2181	8.4	15,073	2.9	536	13.4	2359	3.0
Any other disorder ^‡^	4468	15.6	44,290	7.7	4516	17.5	41,020	7.9	849	21.3	6142	7.7
Violent criminality ^‡^	3619	12.7	16,459	2.9	2400	9.3	16,095	3.1	662	16.6	2521	3.2
Child–parent separation ^‡^	13,053	45.6	162,190	28.4	11,441	44.3	148,400	28.7	2006	50.3	22,779	28.6

^‡^ = occurring between birth and 15th birthday; ^ƚ^ = as recorded at 15th birthday.

**Table 2:** Exposure prevalence by number of childhood adversity risk factors among cases vs. controls for the 3 behavioral outcome categories.

Number of Childhood and Parental/Family Risk Factors	1. Violent Crime (but Not Self-Harm)				2. Self-Harm (but Not Violent Crime)				3. Dual Harm			
	Cases		Controls		Cases		Controls		Cases		Controls	
	(*N* = 28,596)		(*N* = 571,920)		(*N* = 25,826)		(*N* = 516,520)		(*N* = 3987)		(*N* = 79,740)	
	*n*	%	*n*	%	*n*	%	*n*	%	*n*	%	*n*	%
None	5579	19.5	239,005	41.8	5732	22.2	216,667	42.0	524	13.1	32,857	41.2
1	7875	27.5	184,573	32.3	7099	27.5	166,376	32.2	893	22.4	25,589	32.1
2	5940	20.8	83,564	14.6	5145	19.9	74,837	14.5	820	20.6	11,951	15.0
3	3739	13.1	34,288	6.0	3164	12.3	30,556	5.9	566	14.2	4926	6.2
4	2218	7.8	15,210	2.7	1867	7.2	14,026	2.7	413	10.4	2142	2.7
5 or more	3245	11.4	15,280	2.7	2819	10.9	14,058	2.7	771	19.3	2275	2.9

**Table 3:** Incidence rate ratios for adverse childhood experiences (reported in Table 1) across the 3 behavioral outcome categories.

Childhood Adversities	IRR (95% CI)		
	1. Violent Crime (Not Self-Harm)	2. Self-Harm (Not Violent Crime)	3. Dual Harm
**Personal risk factors:**			
Hospitalization due to			
Self-harm ^‡^	2.92 (2.64, 3.23)	5.83 (5.41, 6.28)	6.12 (5.02, 7.46)
Assault ^‡^	4.90 (4.14, 5.79)	4.04 (3.29, 4.97)	7.42 (5.31, 10.38)
Serious accident ^‡^	1.52 (1.47, 1.57)	1.51 (1.46, 1.57)	1.83 (1.69, 1.99)
**Parent/family risk factors:**			
Sociodemographic factors			
Unemployment ^ƚ^	3.77 (3.63, 3.91)	3.20 (3.06, 3.33)	5.15 (4.71, 5.64)
Low educational attainment - both parents ^ƚ^	2.73 (2.66, 2.82)	2.30 (2.23, 2.38)	3.55 (3.29, 3.83)
Younger maternal age	3.16 (3.02, 3.31)	2.65 (2.51, 2.79)	3.94 (3.54, 4.39)
Older paternal age	0.85 (0.81, 0.90)	0.96 (0.92, 1.02)	0.84 (0.73, 0.97)
Residential transience ^‡^	3.03 (2.92, 3.13)	2.68 (2.58, 2.79)	4.38 (4.02, 4.76)
Adverse events			
Sibling death ^‡^	1.35 (1.26, 1.45)	1.39 (1.30, 1.50)	1.70 (1.44, 2.01)
Parental death:			
External causes ^‡^	2.86 (2.64, 3.10)	2.59 (2.38, 2.83)	4.37 (3.67, 5.20)
Natural causes ^‡^	1.57 (1.46, 1.68)	1.45 (1.34, 1.56)	1.74 (1.46, 2.06)
All causes	1.98 (1.88, 2.08)	1.80 (1.70, 1.91)	2.57 (2.27, 2.91)
Hospitalization due to			
Self-harm ‡	3.16 (3.01, 3.32)	3.41 (3.25, 3.59)	4.91 (4.40, 5.48)
Assault ‡	4.37 (4.08, 4.67)	3.31 (3.07, 3.57)	5.90 (5.07, 6.86)
Serious accident ^‡^	1.62 (1.57, 1.67)	1.50 (1.46, 1.55)	1.96 (1.81, 2.11)
Mental illness diagnosis			
Substance misuse disorder ^‡^	3.16 (3.03, 3.31)	3.07 (2.93, 3.22)	5.09 (4.61, 5.63)
Any other disorder ^‡^	2.22 (2.15, 2.29)	2.47 (2.39, 2.56)	3.27 (3.01, 3.54)
Violent criminality ^‡^	4.90 (4.72, 5.09)	3.19 (3.05, 3.34)	6.11 (5.57, 6.70)
Child-parent separation ^‡^	3.01 (2.93, 3.10)	2.57 (2.50, 2.65)	4.58 (4.23, 4.96)

^‡^ = between birth and 15th birthday; ^ƚ^ = at 15th birthday; IRR = Incidence Rate Ratio; CI = Confidence interval.

## Supplementary Materials

The following are available online at https://www.mdpi.com/1660-4601/17/24/9409/s1, Box S1: Coding algorithm to extract information on hospital-treated self-harm. References [27,28,29] are cited in the supplementary materials.
