# Supplementary material for: Adverse Childhood Experiences and Risk of Subsequently Engaging in Self-Harm and Violence towards Other People—“Dual Harm”
Source: Int J Environ Res Public Health. 2020 Dec 15;17(24):9409. doi: 10.3390/ijerph17249409 (PMC7765390; doi:10.3390/ijerph17249409)
Supplement: Supplementary file 1 [file ijerph-17-09409-s001.pdf]

Supplementary Materials: Adverse childhood experiences and risk of subsequently engaging in self-harm and violence towards other people - 'dual harm'

Box S1. Coding algorithm to extract information on hospital-treated self-harm.

There were two components to this algorithm, according to recording procedures in different time periods. From 1987 to 1993, admissions with a "reason for contact code" of 4 (suicide attempt) in the National Hospital Register [27] were identified as self-harm. From 1994 onwards, self-harm was identified from people meeting at least one of the following criteria in the National Hospital Register or Danish Psychiatric Central Register [28]: 'reason for contact code of 4; any psychiatric diagnosis (*ICD-10* chapter F) and a co-morbid diagnosis of poisoning with medication and biological compounds (*ICD-10* codes T36 to T50) or nonmedical compounds, excluding alcohol and poisoning from food (T52 through T60); any psychiatric disorder (*ICD-10* chapter F) and co-morbid diagnosis reflecting lesions on the forearm, wrist, or hand (*ICD-10* codes S51, S55, S59, S61, S65, or S69); any contact with a hospital because of poisoning with weak or strong analgesics, hypnotics, sedatives, psychoactive drugs, antiepileptics, and antiparkinsonian drugs or carbon monoxide (*ICD-10* codes T39, T42, T43, and T58); and any somatic or psychiatric diagnosis X60 to X84' [29].

27. Lynge, E., J. L. Sandegaard and M. Rebolj. "The danish national patient register." *Scandinavian Journal of Public Health* 39 (2011): 30-33. 10.1177/1403494811401482. <Go to ISI>://WOS:000292984700007.
28. Mors, O., G. P. Perto and P. B. Mortensen. "The danish psychiatric central research register." *Scandinavian Journal of Public Health* 39 (2011): 54-57. 10.1177/1403494810395825. <Go to ISI>://WOS:000292984700013.
29. Nordentoft, M., P. B. Mortensen and C. B. Pedersen. "Absolute risk of suicide after first hospital contact in mental disorder." *Archives of General Psychiatry* 68 (2011): 1058-64. <Go to ISI>://WOS:000295486100010.

Filename: ijerph-1023796-supplementary.docx
Directory: D:\1215\ijerph-1023796-4
Template: C:\Users\MDPI\AppData\Roaming\Microsoft\Templates\Normal.dotm
Title: B
Subject:
Author: MDPI
Keywords:
Comments:
Creation Date: 12/15/2020 12:08:00 AM
Change Number: 5
Last Saved On: 12/15/2020 3:40:00 PM
Last Saved By: MDPI-22
Total Editing Time: 1 Minute
Last Printed On: 12/15/2020 9:55:00 PM
As of Last Complete Printing
Number of Pages: 1
Number of Words: 769 (approx.)
Number of Characters: 4,389 (approx.)
